# Saliva pools for screening of human cytomegalovirus using real-time PCR

**DOI:** 10.1007/s00431-020-03842-x

**Published:** 2020-10-14

**Authors:** Cláudia Fernandes, Augusta Marques, Maria de Jesus Chasqueira, Mónica Cró Braz, Ana Rute Ferreira, Ana Serrão Neto, Cândida Mendes, David Lito, Maria-Favila Menezes, Maria José Sousa, Paulo Paixão

**Affiliations:** 1 Centro de Estudos de Doenças Crónicas, CEDOC, Faculdade de Ciências Médicas|NOVA Medical School, Campo Mártires da Pátria, 130, 1169-056 Lisbon, Portugal; 2 Serviço de Pediatria, Hospital CUF Descobertas, Rua Mário Botas, 1998-018 Lisbon, Portugal; 3 Serviço de Pediatria, Hospital Vila Franca de Xira, Estrada Carlos Lima Costa no. 2, 2600-009 Vila Franca de Xira, Portugal; 4 Centro de Medicina Laboratorial Germano de Sousa, Rua Cupertino de Miranda, 1600-513 Lisbon, Portugal

**Keywords:** HCMV, Newborn, Cost reduction, Pools

## Abstract

Human cytomegalovirus (HCMV) is the leading congenital infection agent in the world. The importance of screening this infection has been debated, as 10–15% of the asymptomatic newborns with HCMV at birth will present late sequelae. The aim of this study was to test the feasibility of using saliva pools from newborns in a screening program for congenital HCMV infection, in two Portuguese hospitals. The screening was based on the use of pools of 10 saliva samples for detection of viral DNA by real-time PCR. Whenever there was a positive pool, the samples were tested individually, and for each positive sample the result was confirmed with a urine sample collected in the first 2 weeks of life. The study involved 1492 newborns. One hundred and fifty pools were screened, with 14 positive results in saliva, but only 10 were confirmed in urine samples, giving a prevalence of congenital HCMV infection in both hospitals of 0.67% (CI95% 0.36 to 1.23%).

*Conclusion*: The overall prevalence of congenital HCMV infection in both hospitals was 0.67%. The use of saliva pools proved to be effective for the screening of this congenital infection, allowing timely screening and confirmation in a large population, with associated cost reduction.

**Table:** 

**What is Known:** • *Newborn screening for HCMV is desirable.* • *Saliva is a good and practical sample.*	
**What is New:** • *The feasibility of using saliva pools for a large-scale screening.* • *The cost reduction of this strategy.*

**Electronic supplementary material:**

The online version of this article (10.1007/s00431-020-03842-x) contains supplementary material, which is available to authorized users.

## Introduction

Human cytomegalovirus (HCMV) is the leading congenital infection agent, with an incidence ranging between 0.2 and 2% worldwide [1, 2]. In industrialized countries, an estimated 0.6 to 0.7% of newborns are congenitally infected with this virus [1, 3–5]. Long-term sequelae of this infection affect more children than others with better-known clinical conditions, such as Down syndrome, foetal alcohol syndrome, or spina Bifida. Ongoing special care these children require for life results in substantial economic burden [6]. Strategies to reduce this burden have been debated in the last two decades, including earlier identification of infection through maternal or newborn screening [5, 7].

Maternal screening during pregnancy is not currently advised, and some concerns are raised. Indeed, not only counselling programs and treatment have not yet been proven effective as some adverse effects can be associated with this screening [8].

Between 85 and 90% of newborns with laboratory-confirmed congenital infection will be asymptomatic at birth or present nonspecific symptoms. However, 10 to 15% of these asymptomatic newborns may develop late sequelae at up to 5 years of age such as neurosensorial hearing loss (SNHL), decreased visual acuity, and progressive neurological changes [9, 10]. As neonatal screening for this infection is not currently implemented, a large number of cases of congenital HCMV infection will not be detected. The only way to detect all cases of congenital infection with this virus would be to routinely screen all newborns in the first 2 weeks of life. The implementation of this procedure would enable the monitoring of children with asymptomatic infection at birth, with proven benefit of an early intervention [11, 12]. However, one of the constraints of implementing routine neonatal screening is the associated cost to perform a diagnostic test for each newborn.

Currently, when a congenital infection is suspected, the diagnosis is performed using urine or saliva samples collected within the first 3 weeks of the newborn’s life. In order to circumvent the elevated cost of the screening, our team previously developed a urine pool methodology for congenital HCMV: based on the fact that the viral shed in the urine is high, it can be detected by real-time PCR even if the positive sample is diluted 1/20. This method, which was shown to have similar sensitivity and specificity to cell culture (reference method), allows a significant reduction in reagent cost and execution time. This approach may be feasible for universal screening of this infection [13]. However, urine collection through a paediatric collection bag is a time-consuming procedure with some additional disadvantages, like skin irritation due the glue used to hold the bag, perineum irritation, or delayed collection due to inadequate diuresis or child stress, [14, 15]. Therefore, this collection method may cause discomfort to the child and consequently to parents, preventing its use in a universal screening program.

The possibility of implementing a screening program using saliva samples has been studied in recent years, as saliva is much simpler to collect [16–19]. A recent study in our laboratory has shown that the real-time PCR technique using saliva pools has high sensitivity and specificity (100%) when compared with single sample testing and could easily be applied to large-scale studies, enabling the identification of most newborns with congenital HCMV infection at a significantly reduced cost [20].

The aim of this study was to test the feasibility of using saliva samples from newborns and a pool methodology for a screening program. Secondly, we intended to estimate the prevalence of HCMV congenital infection in two Portuguese hospitals.

## Materials and methods

### Population

The study population included 1492 newborns aged between 1 day and 2 weeks, of which 748 and 744 were born respectively at Hospital CUF Descobertas and at Hospital Vila Franca de Xira (Portugal). The screening was conducted for 8 months, and samples were collected between October 2018 and May 2019.

### Samples

Saliva samples were collected using breakable rod rayon swabs (FLmedical, Torreglia, Italy) and were always carried out before breastfeeding or at least 1 h after the last meal. The swab was packaged in a 3-mL tube with 500-μL RPMI medium (Life Technologies, Paisley, UK). After collection the tubes were stored at 4 °C until processing. Urine samples were requested to confirm congenital infection if a positive result was obtained with a saliva sample. These were collected using paediatric urine collection bags, then placed in 50-mL flasks and stored at 4 °C until processing.

All samples were sent to the Unit of Infection of the Faculdade de Ciências Médicas|Nova Medical School (FCM|NMS) for processing.

### Sample processing

**Fig. 1:**
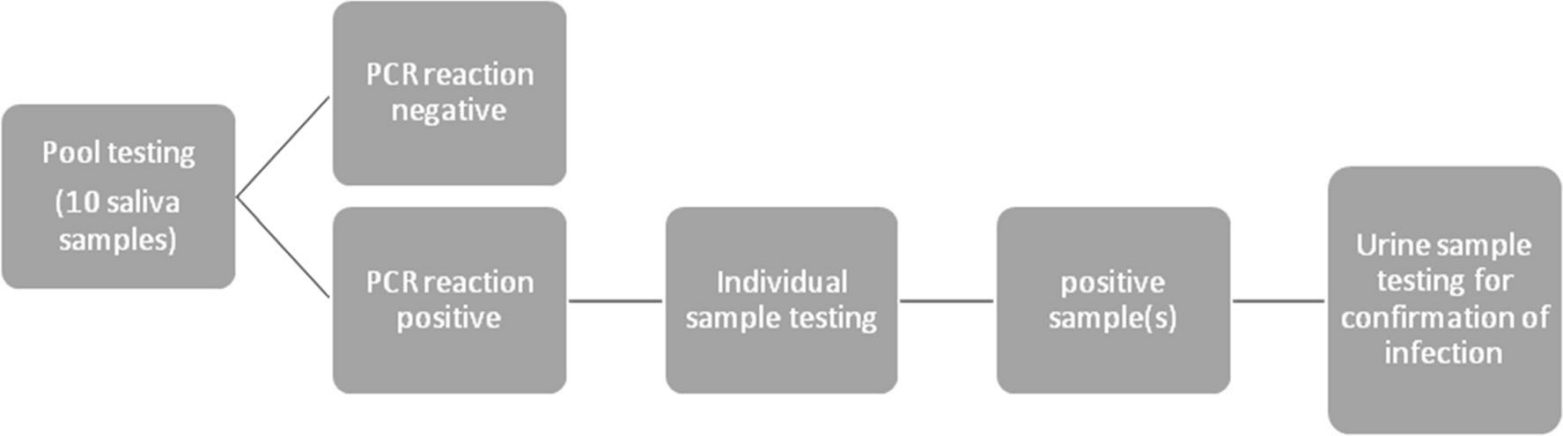
Algorithm used for detection and identification of HCMV DNA positive specimens in 10–-pool saliva samples. When positive, each sample of the pool was tested individually. Upon identification of the positive sample(s) in order to confirm congenital HCMV infection, a urine sample was requested and tested by the same technique as the saliva sample

The samples were centrifuged at 948×*g* (Hettich, Routine 380 R) for 5 min and transferred in a biosafety chamber into properly identified freezing tubes. Disinfection procedures were performed between each sample transfer and every five samples; the work area and material were submitted to UV radiation to avoid contamination. Pools were prepared mixing 20 μL of each 10 saliva samples in a 1.5-mL eppendorf tube. Genomic DNA was extracted using the Purelink Genomic DNA commercial kit (Invitrogen, Carlsbad, USA), according to the manufacturer’s instructions and stored at − 20 °C until use. HCMV DNA was amplified and detected by qPCR on the Applied Biosystems 7500 Fast Real-Time PCR System (Applied Biosystems, Foster City, USA) using an “in-house” method, detailed elsewhere, with 100% sensitivity and specificity when compared with individual samples [13, 20]. Each pool was tested according to the algorithm described in Fig. 1.

Positive and negative controls were added in each series. An internal control was made for each pool and sample tested by adding 2 μL of AD169 strain DNA (69,000 copies/mL) to test for the presence of inhibitory substances. All pools and samples were tested in duplicated.

Quantitative analysis of pools and individual positive samples of saliva and urine were performed using a calibration curve made with four 1:10 serial dilutions of the 1st World Health Organization International Standard, NIBSC 09/162, for HCMV nucleic acid amplification techniques with an initial concentration of 5 × 10^6^ International Units per millilitre (IU/mL). Quantifications were obtained by extrapolating the CT results into the calibration curve, and the results were expressed in IU/mL.

## Results

In this study we grouped 1492 saliva samples into 150 pools of 10 samples each. Of these, 140 tested negatives. Ten positive pools were obtained and, after individual testing of each sample that composed them (following the algorithm described in Fig. 1), we identified 14 positive saliva samples (Table 1). Four pools had two positive samples simultaneously.

**Table 1:** Real-time PCR qualitative results of saliva pools and positive individual samples

10-pool		Positive saliva samples	
No.	UI/mL	No.	UI/mL
POOL 7	1.7 × 10^3^	CMV12A	7.4 × 10^3^
		CMV21A	7.4 × 10^1^
POOL 13	1.8 × 10^6^	CMV82	1.5 × 10^6^
POOL 15	1.2 × 10^7^	CMV57A	8.2 × 10^7^
		CMV58A	1.3 × 10^3^
POOL 21	5.2 × 10^3^	CMV103A	4.2 × 10^4^
POOL 51	6.0 × 10^5^	CMV253	8.4 × 10^5^
POOL 57	1.3 × 10^5^	CMV305A	6.1 × 10^4^
		CMV307A	2.0 × 10^6^
POOL 78	2.5 × 10^3^	CMV420A	1.8 × 10^1^
		CMV422A	8.8 × 10^4^
POOL 90	7.7 × 10^2^	CMV481A	6.9 × 10^3^
POOL 106	4.4 × 10^4^	CMV566A	6.9 × 10^5^
POOL 131	6.8 × 10^3^	CMV633	3.1 × 10^4^

In order to verify the agreement between the methodology used to perform this screening and the reference method (individual testing), samples from 10 pools with negative result were tested individually and obtained concordant results in all samples analysed (100% agreement with the reference test).

For the confirmatory diagnosis, urine samples were requested from the newborns who tested positive with the saliva sample. Of the 14 positive saliva samples, 10 had congenital infection confirmed by the urine analysis and the remaining four were false positive results. These false positive saliva samples had significantly lower HCMV viral loads than the other samples associated with congenital infection (Table 2). Therefore, of the 1492 newborns screened, 10 had confirmed congenital infection, which represents a prevalence of 0.67% in the two hospitals under study (95% confidence interval, exact binomial method: 0.32–1.23%).

**Table 2:** Quantification values in IU/mL for individual positive saliva samples and corresponding 20 sample pools (NT – not tested; Neg – negative)

Positive saliva samples	20-pool
No.	UI/mL
CMV57A	1.2 × 10^7^
CMV82	2.3 × 10^6^
CMV566A	5.3 × 10^5^
CMV253	1.7 × 10^5^
CMV307A	8.1 × 10^4^
CMV422A	1.3 × 10^4^
CMV305A	7.8 × 10^3^
CMV633	2.6 × 10^3^
CMV103A	2.5 × 10^3^
CMV481A	4.7 × 10^2^
CMV12A	4.4 × 10^2^
CMV58A	NT
CMV21A	Neg
CMV420A	Neg

## Discussion

In a nationwide study performed in 2004, the prevalence of HCMV congenital infection in Portugal was described as 1.05%, higher than the prevalence in other European countries [13]. In the current study we found a prevalence of 0.67%, a value similar to the birth prevalence of congenital CMV infection described for developed countries [1, 3–5]. Although the present study was limited to two hospitals in Lisbon and had a low number of samples (giving a 95% confidence interval of 0.32–1.23%), it is possible that the prevalence has been declining in the past years. In 2011, a Portuguese study by Lopo et al. found a lower seroprevalence for HCMV in the central coastal zone, corresponding with our study population; this can explain our results. [21].

Pool methodology for urine samples was proven accurate in a previous study [13]. However, collecting a saliva sample is simpler, faster, and less uncomfortable for the newborns, and therefore better suited for a universal screening program. Concerns about false positive results with these samples have been raised because virus transmission during breastfeeding may occur. Several articles reported the excretion of this virus in the breast milk of healthy women [22, 23]. To prevent these false-positive during screening programs an approach would be to instruct health professionals to collect the sample before the first breastfeeding, although this is not always possible. Therefore, the confirmation of a positive saliva sample should always be performed by viral detection in a urine sample within the first 2 weeks of life [24], which was done with all the cases in our study. This is, however, the general rule for all the saliva samples, regardless if they are tested individually or if first screened by a pool method. In this study, four false positive results were detected with our saliva pool methodology (i.e., the urine samples asked for confirmation were negative in these four children). These false-positive samples had significantly lower HCMV viral loads than the other samples associated with congenital infection, which is in agreement with another study [22], suggesting a dilution effect of the breast milk on the newborn’s saliva and eventually a lower viral load on breast milk. Therefore, the low viral load of these samples reinforces the idea that these saliva-positive/urine-negative samples were in fact false-positive saliva results and not false-negative urine results. Nevertheless, these false-positive results were not related with the pool approach but rather, as explained before, by using saliva instead of urine samples.

Although the reduction in reagent cost and execution time by the pool method was not quantified in the Results section, the advantage of this strategy is obvious: ten samples are screened with a single PCR, instead of testing ten individual samples. With a prevalence around 0.7%, that means that only 1–2 pools will be positive in 20 tested pools, which means that only 30–40 PCR tests will be need to screen 200 newborns, thus reducing the associated cost of this screening when compared with 200 individual tests by the conventional approach. The reason for choosing pools of 10 instead of 20 samples in the current study was a practical decision, to have a faster answer with the pool strategy (results should be ready within 1 week, allowing the confirmatory urine sample to be collected within the first 2 weeks of life). In fact, the 10-pool approach can be used either in hospitals with a few hundred deliveries a year or in hospitals with thousands of newborns. For example, in a hospital with 500–600 births/year, we would have to wait 2 weeks to collect 20 samples for the 20-pool strategy, which would prevent us from giving an answer within the 1-week period indicated above and to get the urine sample for confirmation within the first 2 weeks.

Screening for HCMV infection in pregnancy is not advised for various reasons, such as lack of effective treatment programs and the difficult prognosis regarding the congenital infection sequelae [8]. Thus, only through a universal screening program for congenital HCMV infection in newborns would it be possible to diagnose all cases of infection, especially in the asymptomatic ones. Routine implementation of this procedure would enable early monitoring of children with asymptomatic birth infection, particularly regarding the risk of developing late sequelae such as SNHL and progressive neurological changes. It has been proven that this early intervention benefits quality of life of these children [5, 11, 25].

## Conclusions

Using this saliva pool methodology, we were able to screen 1492 newborns for an 8-month period, with a significant reduction in reagent cost and execution time. These results, combined with the lack of an effective vaccine, open the possibility of using this approach on a large-scale screening for HCMV congenital infection, allowing an early intervention which can benefit the affected children’s life quality. Nevertheless, the feasibility of this approach needs to be confirmed by a larger multicenter study, which is currently underway.

## Electronic supplementary material

ESM 1 (PDF 237 kb)

## Data Availability

The datasets used and/or analysed during the current study are available from the corresponding author on request.

## Abbreviations

HCMV: Human cytomegalovirus
SNHL: Sensorineural hearing loss
