# Supplementary material for: Saliva pools for screening of human cytomegalovirus using real-time PCR
Source: Eur J Pediatr. 2020 Oct 14;180(4):1067–72. doi: 10.1007/s00431-020-03842-x (PMC7940150; doi:10.1007/s00431-020-03842-x)
Supplement: Supplementary file 1 — (PDF 237 kb) [file 431_2020_3842_MOESM1_ESM.pdf]

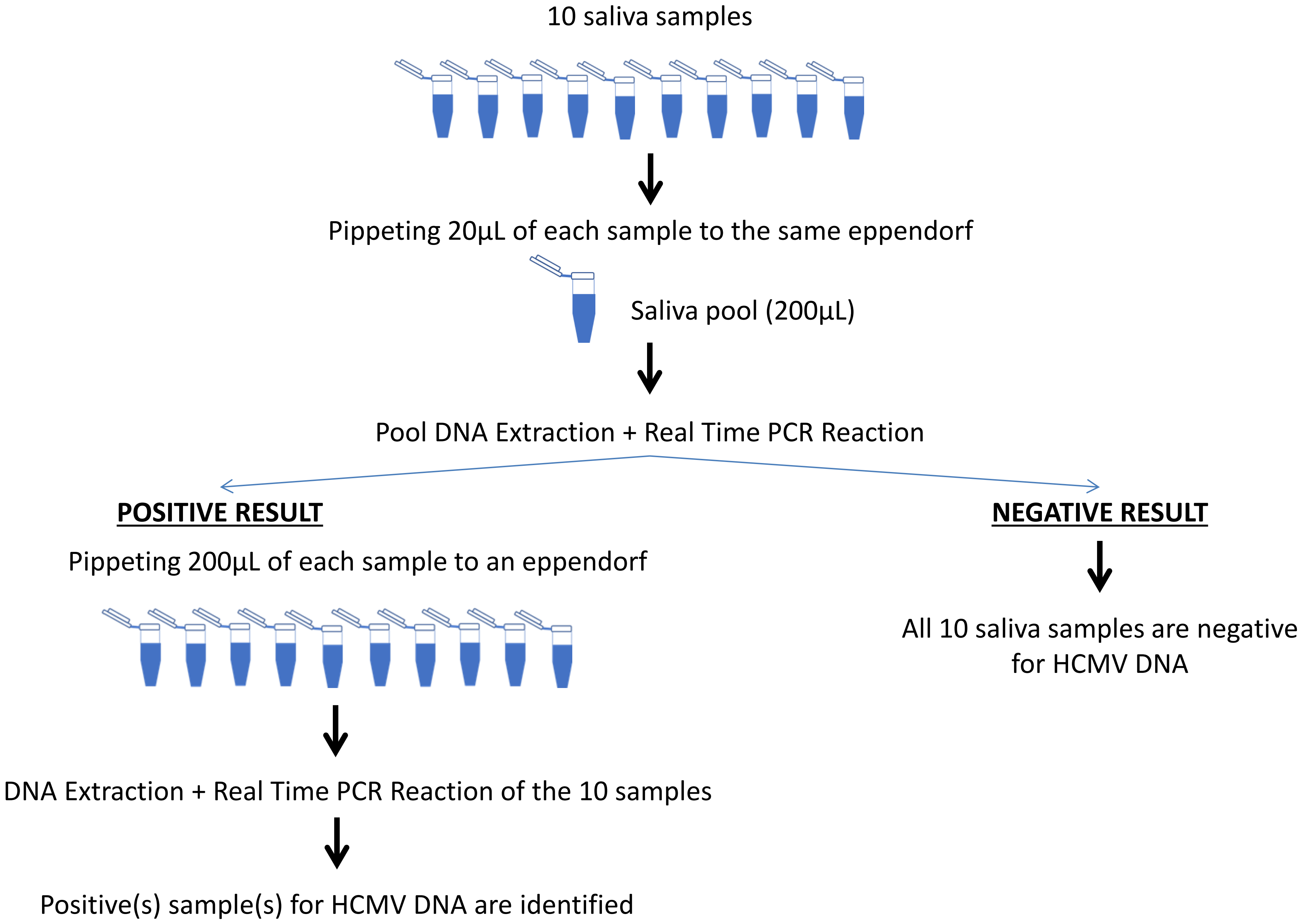
10 saliva samples
Pippeting 20µL of each sample to the same eppendorf
Saliva pool (200µL)
Pool DNA Extraction + Real Time PCR Reaction
POSITIVE RESULT
NEGATIVE RESULT
Pippeting 200µL of each sample to an eppendorf
All 10 saliva samples are negative for HCMV DNA
DNA Extraction + Real Time PCR Reaction of the 10 samples
Positive(s) sample(s) for HCMV DNA are identified
